# Age-dependent interaction between serum zinc and triglyceride-glucose index among American adults: National Health and Nutrition Examination Survey

**DOI:** 10.3389/fnut.2024.1475204

**Published:** 2025-01-13

**Authors:** Jun Lai, Xin-Qing Li, Yinglin Zheng, Zongyan Liu, Qiquan Wu, Yongxiao Cao

**Affiliations:** ^1^Department of Pharmacology, School of Basic Medical Sciences, Xi'an Jiaotong University Health Science Center, Xi'an, Shanxi, China; ^2^Department of Pharmacy, The Affiliated Ganzhou Hospital of Nanchang University, Ganzhou, Jiangxi, China; ^3^Department of Endocrinology, The Affiliated Ganzhou Hospital of Nanchang University, Ganzhou, Jiangxi, China

**Keywords:** serum zinc, triglyceride-glucose index, age, NHANES, cross-sectional analysis

## Abstract

**Introduction:**

Zinc plays a crucial role in glucose metabolism. The association between serum zinc and insulin resistance has recently been investigated as well, but the findings are inconsistent. The triglyceride-glucose index (TyG) is frequently utilized in epidemiological research to assess insulin resistance. The association between serum zinc levels and TyG has not yet been explored. Therefore, we designed this cross-sectional study to assess the relationship between serum zinc and TyG in adults using data from the National Health and Nutrition Examination Survey (NHANES).

**Methods:**

A cross-sectional analysis was performed on 1,610 adults aged ≥20 years who participated in the National Health and Nutrition Examination Survey (NHANES) 2011–2016. The participants were stratified by age, and the differences in log-transformed serum zinc quartiles and TyG were further evaluated in age groups <60 years and ≥60 years using multivariable linear regression with an interaction test. Additionally, a restricted cubic spline (RCS) model was employed to examine the dose-response relationships between log-transformed serum zinc and TyG.

**Results:**

In this cross-sectional study, a significant interaction was observed between log-transformed serum zinc and TyG in individuals aged <60 years and those aged ≥60 years when log-transformed serum zinc was transformed into a categorical variable (*P*-value for the likelihood ratio test for the interaction was *P* = 0.017). Additionally, in the fully adjusted analyses, the association between log-transformed serum zinc and TyG in the age <60 years group demonstrated a J-shaped nonlinear pattern (*P* for nonlinearity = 0.014), with an inflection point at ~1.94 μg/dL. While in the age ≥60 years group, it exhibited an inverted-L shaped nonlinear pattern (*P* for nonlinearity < 0.001^***^).

**Conclusion:**

There is a significant relationship between log-transformed serum zinc and TyG in adults in the United States, with age potentially influencing this association. Further prospective studies are needed to offer additional evidence and insights into these findings.

## 1 Introduction

Zinc, the second most prevalent trace metal in the human body, is a vital micronutrient essential for growth and development (1). It is a component of numerous enzymes (2) and may play a protective role by regulating inflammation, reducing oxidative stress, and participating in lipid and glucose metabolism (3). Additionally, zinc is crucial in the biochemistry of insulin and glucagon within pancreatic β- and α-cells (4), playing a key role in the synthesis, storage, and release of insulin, and is linked to diabetes and metabolic syndrome (3, 5). Over recent decades, zinc has been extensively studied for its antioxidative and anti-inflammatory properties. Mild or moderate zinc deficiency in humans can result in stunted growth, delayed puberty in adolescents, hypogonadism in males, dermatitis, decreased appetite, mental lethargy, and delayed wound healing (6). However, several studies showed high doses of zinc-based biomaterials may have adverse effects, including liver, spleen, and pancreas damage in mice, disruption of energy metabolism, and impairment of mitochondrial and cell membrane function in rat kidneys (7–9). Assessing zinc status is challenging due to tightly regulated zinc homeostasis. The Biomarkers of Nutrition for Development Zinc Expert Panel and the International Zinc Nutrition Consultative Group recommend using plasma or serum zinc concentration as a biomarker for zinc status (10).

Insulin resistance is closely linked to risk factors for cardiovascular and metabolic diseases, including coronary heart disease, stroke, hypertension, atherosclerosis, diabetes, and atrial fibrillation (11–13). It significantly contributes to the morbidity and mortality rates associated with these conditions, as well as imposing a substantial economic burden (14). Currently, the hyperinsulinemic-euglycemic clamp (HEC) is considered the gold standard for evaluating insulin sensitivity in peripheral tissues (15). However, this invasive method is complex, time-consuming, and technically challenging, which has led to a preference for simpler indicators of insulin resistance. Traditional measures like the homeostatic model assessment for insulin resistance (HOMA-IR) and the quantitative insulin sensitivity check index (QUICKI), both of which rely on fasting insulin levels, are limited by practical constraints and variability (16). The triglyceride-glucose index (TyG) is a reliable and easily acquired indicator which is derived from fasting plasma glucose and triglyceride (TG) levels, serves as an indicator for assessing insulin resistance in epidemiological research (17). TyG has emerged as a novel tool that demonstrates superiority over HOMA-IR in evaluating insulin resistance, particularly in individuals with diabetes undergoing insulin therapy or those lacking functional beta cells (18–21). A 12-year longitudinal study from the Korean Genome and Epidemiology Study cohort found that a higher TyG index precedes and significantly predicts type 2 diabetes in community-dwelling, middle-aged, and elderly lean Koreans (22). Several studies have provided evidences linking TyG to the onset and prognosis of cardiovascular diseases, including stable coronary artery disease, carotid plaque, coronary artery calcification, and acute coronary syndrome (13, 23–25). Moreover, TyG is closely associated with cardiovascular disease risk factors such as arterial stiffness and hypertension (11, 12).

The association between serum zinc and insulin resistance has recently been investigated as well, but the findings are inconsistent. Some studies have documented that zinc deficiency may predispose glucose intolerance and insulin resistance, diabetes mellitus, and coronary artery disease (26–29). While previous studies suggest that higher serum zinc concentrations may be associated with an increased risk of metabolic syndrome (5, 30, 31), and serum zinc concentration was significantly higher in both abnormal glucose tolerance and the presence of diabetes individuals (32). Animal research shows that the administration of zinc in small doses has been demonstrated to confer protection against type 2 diabetes; however, a high concentration of the element has been shown to exert a toxic effect on the beta cells within the islets of Langerhans (33). Meanwhile, a study found statistically significant positive association between zinc and HOMA-IR in men aged 50–75 years without diabetes, and the men with metabolic syndrome showed statistically significant higher zinc (34). But a study report that there is no statistically significant association between the concentration of zinc and metabolic syndrome with its individual components in adults from Lebanon aged 18–65 years (35).

Our study aims to examine the association between serum zinc levels and TyG, which has not yet been explored. To fill this knowledge gap, we evaluated the relationship between serum zinc and TyG in adults using data from the National Health and Nutrition Examination Survey (NHANES). Our hypothesis was that individuals with elevated TyG levels would have higher serum zinc levels, based on observed nutritional patterns in this population. Additionally, we assessed the dose-response relationship between serum zinc and TyG.

## 2 Materials and methods

### 2.1 Data sources and study population

The National Health and Nutrition Examination Survey (NHANES) is a series of health-related research aimed at determining non-institutionalized Americans' health and nutritional status. As a representative sample, a multistage, stratified probability strategy was used to select survey participants (36). This cross-sectional study used the data from 2011–2012, 2013–2014, and 2015–2016 cycles from the NHANES, as the interesting trace metal was only examined in these three survey waves. Demographic, socioeconomic and health-related information were collected through questionnaires, physical examinations, and laboratory tests. Health interviews were conducted at participants' homes, while thorough physical examinations, including blood sample collection, were carried out at the Mobile Examination Center (MEC). The collected serum specimens were then tested at the National Center for Environmental Health's Division of Laboratory Sciences of the Centers for Disease Control and Prevention (37).

The NHANES was authorized by the National Center for Health Statistics Ethics Review Board (https://www.cdc.gov/nchs/nhanes/irba98.htm). Before participating, all participants completed written informed consent forms. The secondary analysis did not require additional Institutional Review Board approval (38). The NHANES data are accessible through the NHANES website (http://www.cdc.gov/nchs/nhanes.htm; accessed on 19 Oct 2023).

### 2.2 Inclusion criteria

Our study's participants were above the age of 20 and had completed an interview and evaluation at a MEC.

### 2.3 Exclusion criteria

We excluded pregnant women or individuals with missing data on serum zinc, fasting plasma glucose (FPG), triglyceride (TG) or covariates. And we excluded participants with extreme energy intake, consuming <500 or >5,000 kcal per day.

### 2.4 Serum zinc

Serum zinc was detected at the Environmental Health Sciences Laboratory of the CDC National Center for Environmental Health using the inductively coupled plasma dynamic reaction cell mass spectrometry following extensive quality control procedures. The lower limit of detection (LLOD) for serum zinc was 2.9 μg/dL, and all the data was above the LLOD for all tests. In the multivariable linear models, log-transformed serum zinc was categorized into quartiles: Q1 (1.69–1.89 μg/dL; *n* = 397), Q2 (1.90–1.93 μg/dL; *n* = 408), Q3 (1.94–1.97 μg/dL; *n* = 401), Q4 (1.98–2.37 μg/dL; *n* = 404).

### 2.5 Triglyceride-glucose index

Triglyceride-glucose index (TyG) was calculated using the formula Ln [fasting TG (mg/dL) × fasting plasma glucose (FPG; mg/dL)/2] (18). Blood samples were taken in the morning after fasting overnight to measure the levels of TG and glucose in the blood. The concentration of TG and FPG was measured using an automatic biochemistry analyzer. The serum TG levels were determined using a Roche Cobas 6000 chemistry analyzer and a Roche Modular P chemistry analyzer. A Roche/Hitachi Cobas C 501 chemistry analyzer was used to measure FPG using the hexokinase-mediated reaction.

### 2.6 Covariates

The covariates considered in this study consisted of sociodemographic, behavioral, health characteristics and laboratory data deemed a priori as potential confounders.

Sociodemographic variables consisted of age groups (20–59 years and ≥60 years) (39–41), gender (female and male), race/ethnicity (non-Hispanic White, non-Hispanic Black, Mexican American, or other races), education level (<9, 9–12, or >12 years), marital status (married, living with a partner, or living alone). According to a US government report (42), family income was categorized into three groups by the poverty income ratio (PIR): low (PIR ≤ 1.3), medium (PIR > 1.3–3.5), and high (PIR > 3.5).

Behavioral characteristics comprised smoking status, drinking status, and physical activity. According to previous literature definitions (43), smoking status was classified into three categories: never smokers (participants who had smoked fewer than 100 cigarettes), current smokers, and former smokers (those who had quit smoking after smoking more than 100 cigarettes). Furthermore, individuals who consumed at least 12 alcoholic drinks per year throughout their lifetime were classified as drinkers (37). Physical activity was categorized as sedentary, moderate (involving at least 10 min of movement within the past 30 days, resulting in light sweating or a mild to moderate increase in breathing or heart rate), and vigorous (involving at least 10 min of activity within the past 30 days, resulting in profuse sweating or a significant increase in breathing or heart rate) (43).

Health factors included body mass index (BMI), trouble sleeping, hypertension (no or yes) (44), diabetes (no or yes) (45) and failing kidneys (no or yes) (46). BMI was computed using a standardized technique which is weight (kg) divided by height (m) and divided into four categories with cut-off values of 18.5, 25, and 30 kg/m^2^ (underweight, normal, overweight, and obese) (47). Hypertension was diagnosed based on a self-reported physician diagnosis (a positive response to “Have you been diagnosed with hypertension?”), and/or recent use of an antihypertensive agent (a positive response to “Are you currently taking any antihypertensive drugs to treat or control your blood pressure?”), and/or a systolic blood pressure/diastolic blood pressure ≥140/90 mmHg (44). Diabetes cases were defined as participants who fulfilled the inclusion criteria: (1) FPG ≥126 mg/dL, (2) 2-h plasma glucose ≥200 mg/dL on an oral glucose tolerance test (OGTT), (3) HbA1c ≥6.5%, and (4) current use of insulin or diabetes pills to lower blood glucose levels, or a self-report questionnaire that indicates a previously diagnosed of T2DM by a physician (45). Failing kidneys was determined for participants who positively responded to the question has he/she ever been told by a doctor or other health professional that had weak or failing kidneys (excluding kidney stones, bladder infections, or incontinence) (46). A dietary recall interview preceded and interview including total energy intake.

Laboratory data including HbA1c, high density lipoprotein cholesterol (HDL-C), triglyceride, creatinine, total cholesterol, FPG and uric acid.

### 2.7 Statistical analysis

Statistical analyses were performed using the statistical software programs R (The R Foundation) and Free Statistics software version 1.9.2 (Beijing Free Clinical Medical Technology Co., Ltd.) (48). All statistical tests were two-sided, and significance was considered at *P* < 0.05. Analyses were conducted according to the Centers for Disease Control and Prevention (CDC) guidelines for the analysis of NHANES data. As the sample size was determined based solely on the available data, no a priori statistical power estimates were conducted. We used fasting subsample MEC weights for the weighted analysis. For the combined analyses of NHANES 2011–2016 data, a 6-year fasting subsample MEC weights (WTSAF2YR) set was used, stratum (SDMVSTRA), and primary sampling units (SDMVPSU) were taken into account for the complex survey design (48).

Categorical data were expressed as unweighted numbers (weighted percentages), whereas continuous data were expressed as means (standard deviation, SD). One-way analyses of variance (continuous variables) and chi-square tests (categorical variables) were used to compare differences between the groups. To analyze the association between serum zinc and TyG, we used univariate and multivariable linear regression models. The models integrated regression coefficients (β) and 95% confidence intervals (CI) while controlling for significant covariates. Log-transformed serum zinc was considered a continuous variable after undergoing a logarithm 10 transformation. The selection of confounding variables was guided by clinical relevance, existing scientific literature, the significance of covariates in univariate analysis, their correlation with the outcomes of interest, or a change in effect estimate exceeding 10%. In multivariable linear regression, we showed (1) unadjusted models, (2) model 1 adjusted covariates with a change in effect estimate exceeding 10%, including sex, BMI, HDL-C, TC, uric acid, diabetes and trouble sleeping, (3) model 2 adjusted for variables from model 1 plus covariates that *P* values were < 0.05 in the univariate analysis, including age, race and ethnicity, educational level, physical activity, smoking status, HbA1c, failing kidneys, hypertension, and (4) model 3 adjusted for variables from model 2 plus covariates that on the basis of previous findings and clinical constraints, including marital status, PIR, drinking status, creatinine, total energy intake (17, 49).

In addition, we examined possible dose-response relationships between log-transformed serum zinc and TyG after adjusting variables in model 3, restricted cubic spline (RCS) regression was performed with 4 knots at the 5th, 35th, 65th, and 95th percentiles of the distribution (43).

We used a two-piece-wise linear regression model with smoothing to analyze the association threshold between log-transformed serum zinc and TyG after adjusting the variables in model 3, the likelihood-ratio test and the bootstrap resampling method were used in determining inflection points, in addition to conducting separate analyses for the age groups <60 years and age ≥60 years.

Furthermore, we compared potential modifications of the relationship between log-transformed serum zinc and TyG in the groups with age <60 years and age ≥60 years. The heterogeneity in the subgroup were assessed using multivariable linear regression and interactions between the subgroup and log-transformed serum zinc were examined through likelihood ratio testing.

## 3 Results

### 3.1 Study population

**Figure 1:**
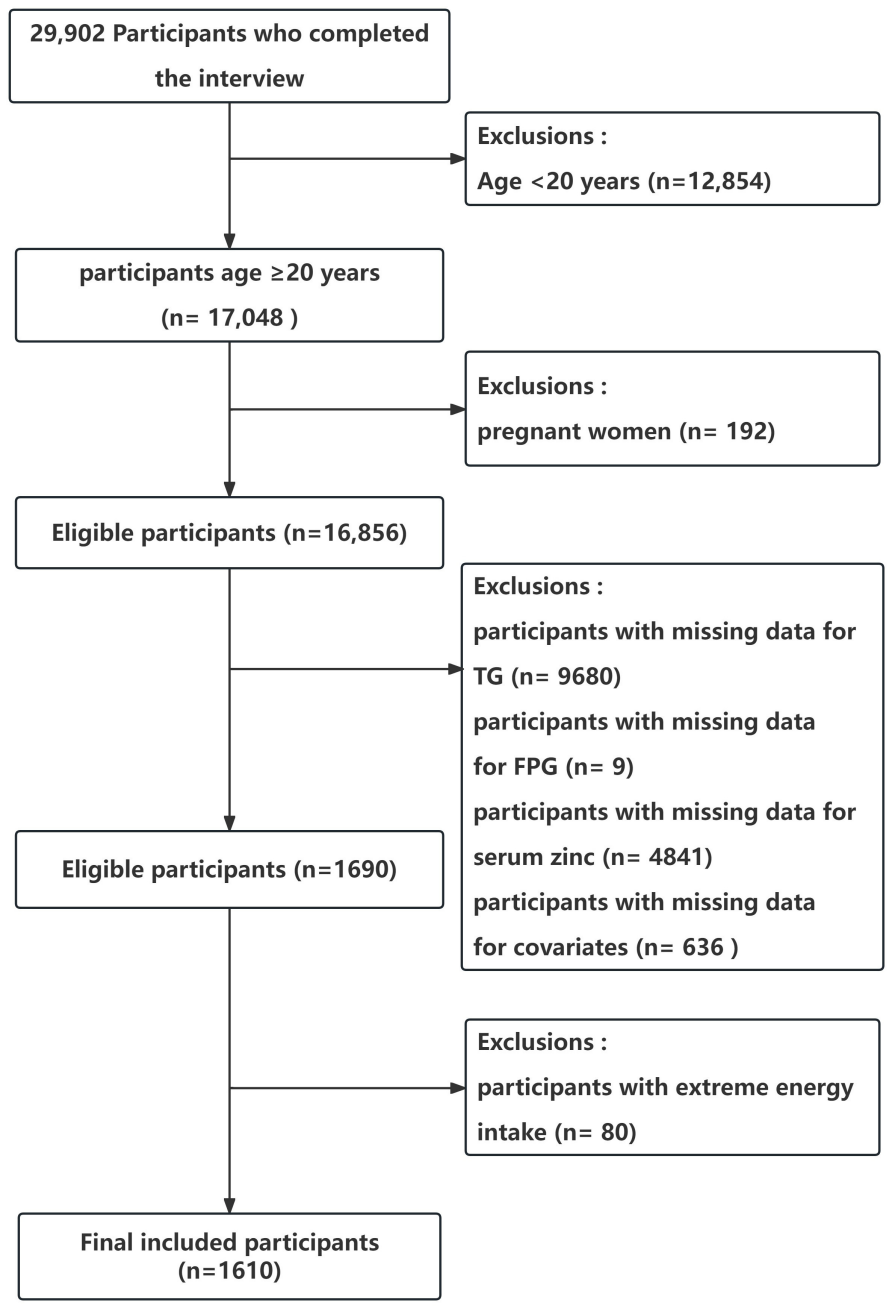
Flow chart of the study population enrollment. TG, triglyceride; FPG, fasting blood glucose.

In total, 29 902 participants completed the interview, of whom 12,854 participants were < 20 years old. We excluded pregnant women (*n* = 192), those missing data on TG (*n* = 9,680), those missing data on FPG (*n* = 9), those missing data on serum zinc (*n* = 4,841), or those with covariates (*n* = 636). And we excluded participants with extreme energy intake, consuming < 500 or >5,000 kcal per day (*n* = 80). Ultimately, this cross-sectional study included 1 610 participants from the NHANES between 2011 and 2016 in the analysis. The detailed inclusion and exclusion process is shown in Figure 1. The figure delineates the study's design, sampling, and exclusion procedures. This study included American adults (aged ≥20 years) who participated in the 2011–2012, 2013–2014, and 2015–2016 cycles of NHANES, as the serum zinc is only assessed during these survey waves.

### 3.2 Baseline characteristics

The Supplementary Table S1 describes the baseline characteristics of the excluded and included participants. Table 1 illustrates the baseline characteristics of all subjects based on their age, categorized into the age < 60 group and age ≥60 group. The average age of the study participants was 49.6 (17.6) years, and 839 (52.1%) individuals were male. In comparison to the age < 60 group, the age ≥60 group exhibited elevated levels of HbA1c, HDL-C, creatinine, FPG, uric acid, and TyG. Furthermore, they demonstrated a higher prevalence of hypertension, diabetes, renal impairment, and sedentary physical activity, along with a lower prevalence of current smoking, current alcohol use, and lower educational attainment. The log-transformed serum zinc levels in the age ≥60 group were comparable to those in the age < 60 group (*P* = 0.29).

**Table 1:** Baseline characteristics of participants.

**Covariates**	**Total (*n* = 1,610)**	**Age < 60 (*n* = 1,065)**	**Age ≥60 (*n* = 545)**	***P*-value**
Age (mean ± SD, years)	49.6 ± 17.6	39.4 ±11.7	69.6 ± 6.6	< 0.001
**Gender**, ***n*** **(%)**				
Male	839 (52.1)	550 (51.6)	289 (53.0)	0.60
Female	771 (47.9)	515 (48.4)	256 (47.0)	
**Race/Ethnicity**, ***n*** **(%)**				
Non-Hispanic White	696 (43.2)	415 (39.0)	281 (51.6)	< 0.001
Non-Hispanic Black	303 (18.8)	209 (19.6)	94 (17.2)	
Mexican American	219 (13.6)	157 (14.7)	62 (11.4)	
Others	392 (24.4)	284 (26.7)	108 (19.82)	
**Education level**, ***n*** **(%)**				
< 9	137 (8.5)	59 (5.5)	78 (14.3)	< 0.001
9–12	556 (34.5)	357 (33.5)	199 (36.5)	
>12	917 (57.0)	649 (60.9)	268 (49.2)	
**Marital status**, ***n*** **(%)**				
Living alone	969 (60.2)	634 (59.5)	335 (61.5)	0.45
Married or living with a partner	641 (39.8)	431 (40.5)	210 (38.5)	
**PIR**, ***n*** **(%)**				
Low (PIR ≤ 1.3)	526 (32.7)	349 (32.8)	177 (32.5)	0.37
Medium (PIR >1.3–3.5)	604 (37.5)	388 (36.4)	216 (39.6)	
High (PIR >3.5)	480 (29.8)	328 (30.8)	152 (27.9)	
BMI (kg/m^2^), mean ± SD	29.3 ± 7.1	29.3 ± 7.4	29.3 ± 6.4	0.93
**BMI**, ***n*** **(%)**				
< 18.5 kg/m^2^	30 (1.9)	23 (2.2)	7 (1.3)	0.33
18.5–24.9 kg/m^2^	442 (27.4)	301 (28.3)	141 (25.9)	
25–29.9 kg/m^2^	526 (32.7)	336 (31.6)	190 (34.9)	
≥30 kg/m^2^	612 (38.0)	405 (38.0)	207 (38.0)	
**Physical activity**, ***n*** **(%)**				
Sedentary	743 (46.2)	452 (42.4)	291 (53.4)	< 0.001
Moderate	537 (33.4)	354 (33.2)	183 (33.6)	
Vigorous	330 (20.5)	259 (24.3)	71 (13.0)	
**Smoking status**, ***n*** **(%)**				
Never	890 (55.3)	625 (587)	265 (48.6)	< 0.001
Former	408 (25.3)	192 (18.0)	216 (39.6)	
Current	312 (19.4)	248 (23.3)	64 (11.7)	
**Drinking status**, ***n*** **(%)**				
≥12 alcohol drinks a year	1,189 (73.8)	816 (76.6)	373 (68.4)	< 0.001
Trouble sleeping, *n* (%)	431 (26.8)	260 (24.4)	171 (31.4)	0.0029
HbA1c (%), mean ± SD	5.77 ± 1.08	5.62 ±1.05	6.07 ± 1.08	< 0.001
HDL-C (mg/dL), mean ± SD	54.0 ± 15.8	52.7 ±14.9	56.5 ±17.3	< 0.001
TG (mg/dL), mean ± SD	114.1 ± 65.4	112.4 ± 67.1	117.4 ±61.8	0.14
Creatinine (mg/dL), mean ± SD	0.89 ± 0.45	0.84 ± 0.36	1.00 ± 0.57	< 0.001
TC (mg/dL), mean ± SD	191 ± 41	191 ± 40	190 ± 41	0.62
Energy (kcal), mean ± SD	2,119 ± 873	2,233 ± 912	1,897 ±744	< 0.001
FPG (mg/dL), mean ± SD	109 ±34	105 ±32	116 ±36	< 0.001
Uric acid (mg/dL), mean ± SD	5.53 ± 1.37	5.45 ± 1.38	5.68 ± 1.35	0.0014
TyG, mean ± SD	8.55 ±0.64	8.49 ± 0.66	8.67 ±0.60	< 0.001
log-transformed serum zinc (μg/dL), mean ± SD	1.94 ±0.07	1.94 ±0.07	1.94 ±0.07	0.29
Hypertension, *n* (%)	748 ±46	386 ±36	362 ±66	< 0.001
Diabetes, *n* (%)	365 (22.7)	144 (13.5)	221 (40.6)	< 0.001
Failing kidneys, *n* (%)	60 (3.7)	22 (2.1)	38 (7.0)	< 0.001

Data presented are mean ± SD or N (%).

SD, standard deviation; PIR, family poverty income ratio; BMl, body mass index; HbA1c, glycated hemoglobin A1c; HDL-C, high density lipoprotein cholesterol; TG, triglyceride; TC, total cholesterol; FPG, fasting blood glucose; TyG, triglyceride-glucose index.

### 3.3 Relationship between serum zinc level and TyG

The univariate analysis demonstrated that age, sex, race/ethnicity, education level, BMI, physical activity, smoking status, trouble sleeping, HbA1c, HDL-C, TC, uric acid, failing kidneys, hypertension, diabetes, and log-transformed serum zinc were associated with TyG (Table 2).

**Table 2:** Association of convariates and triglyceride-glucose index.

**Variable**	**β (95% CI)**	***P*-value**
Age (years)	0.01 (0.01 to 0.01)	< 0.001
**Gender**		
Male	0 (reference)	< 0.001
Female	−0.19 (−0.27 to −0.12)	
**Race/Ethnicity**		
Non-Hispanic White	0 (reference)	
Non-Hispanic Black	−0.26 (−0.36 to −0.15)	< 0.001
Mexican American	0.02 (−0.09 to 0.13)	0.710
Others	−0.05 (−0.13 to 0.03)	0.219
**Education level**		
< 9	0 (reference)	
9–12	−0.09 (−0.23 to 0.05)	0.199
>12	−0.22 (−0.34 to −0.11)	< 0.001
Married or living with a partner	−0.05 (−0.13 to 0.02)	0.154
BMI kg/m^2^	0.03 (0.02 to 0.03)	< 0.001
**BMI**		
< 18.5 kg/m^2^	0 (reference)	
18.5–24.9 kg/m^2^	−0.02 (−0.19 to 0.16)	0.845
25–29.9 kg/m^2^	0.41 (0.22 to 0.59)	< 0.001
≥30 kg/m^2^	0.52 (0.34 to 0.70)	< 0.001
**Physical activity**		
Sedentary	0 (reference)	
Moderate	−0.16 (−0.27 to −0.05)	0.005
Vigorous	−0.10 (−0.21 to 0)	0.053
**Smoking status**		
Never	0 (reference)	
Former	0.13 (0.02 to 0.23)	0.02
Current	0.17 (0.04 to 0.31)	0.014
Drinking status	0 (reference)	
≥12 alcohol drinks a year	0.04 (−0.06 to 0.14)	0.384
Trouble sleeping	0.14 (0.05 to 0.24)	0.004
HbA1c (%)	0.28 (0.23 to 0.34)	< 0.001
HDL (mg/dL)	−0.02 (−0.02 to −0.02)	< 0.001
TC (mg/dL)	0.01 (0 to 0.01)	< 0.001
Creatinine (mg/dL)	0.20 (0.04 to 0.36)	0.107
Uric acid (mg/dL)	0.14 (0.11 to 0.18)	< 0.001
Failing kidneys	0.25 (0.09 to 0.41)	0.003
Hypertension	0.32 (0.25 to 0.40)	< 0.001
Diabetes	0.60 (0.51 to 0.69)	< 0.001
Log-transformed serum zinc (μg/dL)	1.16 (0.55–1.78)	< 0.001

Data presented are β and 95% CI.

BMl, body mass index; HbA1c, glycated hemoglobin A1c; HDL-C, high density lipoprotein cholesterol; TC, total cholesterol; 95% CI, 95% confidence interval.

The findings of the multivariable linear regression analysis are shown in Table 3. In the unadjusted model, there was a positive association of log-transformed serum zinc with TyG (β = 1.16, 95% CI = 0.55–1.78). Results were similar after adjusting for sex, BMI, HDL-C, TC, uric acid, diabetes and trouble sleeping (β = 0.50, 95% CI = 0.04–0.96). After adjusting for other possible confounders, including age, race and ethnicity, educational level, physical activity, smoking status, HbA1c, failing kidneys, hypertension, marital status, PIR, alcohol, creatinine, and total daily energy intake, the positive association remained significant (β = 0.50, 95% CI = 0.08–0.93; *P* < 0.05). When log-transformed serum zinc was analyzed using quartiles, the association between TyG was consistent across all models, indicating their robustness (Table 3). The individuals with quartile 3 (Q3) group of log-transformed serum zinc (1.94–1.97 μg/dL) were used as the baseline reference, those with Q4 group of log-transformed serum zinc (1.98–2.37 μg/dL) had an adjusted β for TyG of 0.092 (95% CI 0.013–0.17, *P* < 0.05; Table 3) after adjusting for the variables in Model 3.

**Table 3:** Multivariable linear regression was used to determine the relationship between log-transformed serum zinc and triglyceride-glucose index, weighted.

**Variable**	**No**.	**Crude model**		**Model 1**		**Model 2**		**Model 3**	
		β **(95% CI)**	***P*** **value**	β **(95% CI)**	***P*** **value**	β **(95% CI)**	***P*** **value**	β **(95% CI)**	***P*** **value**
log-transformed serum zinc (μg/dL)	1,624	1.16 (0.55 to 1.78)	< 0.001	0.50 (0.04 to 0.96)	0.033	0.50 (0.08 to 0.92)	0.022	0.50 (0.08 to 0.93)	0.023
**Quartiles [log-transformed serum zinc (**μ**g/dL)]**									
Q1 (1.69–1.89)	397	−0.07 (−0.19 to 0.06)	0.28	0.004 (−0.08 to 0.09)	0.92	0.004 (−0.08 to 0.09)	0.93	0.01 (−0.08 to 0.09)	0.89
Q2 (1.90–1.93)	408	−0.08 (−0.20 to 0.05)	0.28	0.01 (−0.06 to 0.08)	0.76	0.01 (−0.05 to 0.07)	0.75	0.01 (−0.06 to 0.07)	0.83
Q3 (1.94–1.97)	401	0 (Ref)		0 (Ref)		0 (Ref)		0 (Ref)	
Q4 (1.98–2.37)	404	0.13 (0.02 to 0.23)	0.016	0.09 (0.02 to 0.17)	0.015	0.087 (0.012 to 0.16)	0.025	0.09 (0.01 to 0.17)	0.025
*P* for trend			0.031		0.011		0.018		0.020

Data presented are β and 95% CI.

Model 1: Adjusted for gender, BMI, HDL-C, TC, uric acid, diabetes, trouble sleeping.

Model 2: Adjusted for Model 1 + age, race and ethnicity, educational level, physical activity, smoke, HbA1c, failing kidneys, hypertension.

Model 3: Adjusted for Model 2+ marital status, PIR, alcohol, creatinine, energy.

BMl, body mass index; HbA1c, glycated hemoglobin A1c; HDL-C, high density lipoprotein cholesterol; TC, total cholesterol; PIR, family poverty income ratio; 95% CI, 95% confidence interval.

**Figure 2:**
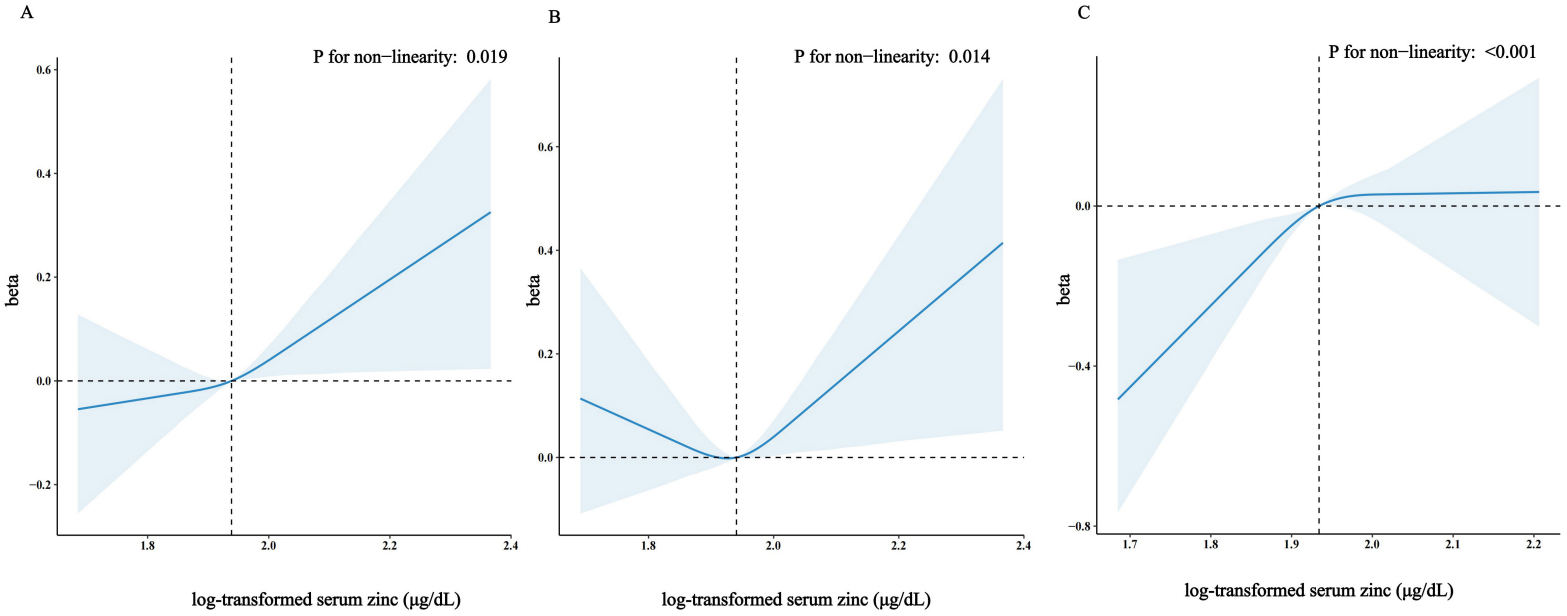
Restricted cubic spline model of the β coefficient of log-transformed serum zinc level with TyG with age ≥20 years **(A)** or with age <60 years **(B)** or with age ≥60 years **(C)**. Adjusted for gender, body mass index (BMI), high-density lipoproteins (HDL), total cholesterol (TC), uric acid, diabetes, trouble sleeping, age, race and ethnicity, educational level, physical activity, smoke, HbA1c, failing kidneys, hypertension, marital status, poverty to income ratio (PIR), drinking status, creatinine, total energy intake. The dashed lines represent the 95% confidence intervals. Heavy central lines represent the estimated adjusted correlation coefficient (β), with LightSkyBlue shaded ribbons denoting 95% confidence intervals. The horizontal dotted lines represent the correlation coefficient (β) of 0 (Reference point). The reference point was set at the median level of log-transformed serum zinc (1.94 μg/dL), and the vertical dotted lines indicate the threshold value of log-transformed serum zinc at 1.94 μg/dL.

Accordingly, in the fully adjusted analyses, the restricted cubic spline (RCS) regression (Figure 2) indicated a non-linear relationship between log-transformed serum zinc and TyG levels in a J-shaped manner (*P* for nonlinearity = 0.019) (A). A segmented regression model was employed to delineate the intervals and calculate threshold effects, with an inflection point at ~1.94 μg/dL. The results are presented in Table 4. When the log-transformed serum zinc was < 1.94 μg/dL, the estimated dose-response curve exhibited a consistent horizontal trend, and the relationships between the log-transformed serum zinc and TyG was not significant (*P* > 0.05). Likewise, the TyG exhibited an increase with rising log-transformed serum zinc after the inflection point, with a correlation coefficient (β) of 0.96 (95% CI: 0.25–1.66) after adjusting for the variables in Model 3 (Table 4). Furthermore, within the age <60 years group, the TyG demonstrated an increase with escalating log-transformed serum zinc after the inflection point, with a correlation coefficient (β) of 1.21 (95% CI: 0.49–1.94) after adjusting for the variables in Model 3. Conversely, within the age ≥60 years group, the TyG exhibited an increase with increasing log-transformed serum zinc prior to the inflection point, with a correlation coefficient (β) of 1.98 (95% CI: 0.62–3.35) after adjusting for the variables in Model 3 (Table 5).

**Table 4:** Association between log-transformed serum zinc level and triglyceride-glucose index using two-piece-wise regression models.

**Variable log-transformed serum zinc (μg/dL)**	**Crude model**		**Adjusted model**	
	β **(95% CI)**	***P*** **value**	β **(95% CI)**	***P*** **value**
< 1.94	0.33 (−0.85 to 1.50)	0.58	0.26 (−0.65 to 1.16)	0.56
≥1.94	1.52 (0.34 to 2.70)	0.013	0.96 (0.25 to 1.66)	0.01

Data presented are β and 95% CI.

Adjusted for gender, BMI, HDL-C, TC, uric acid, diabetes, trouble sleeping, age, race and ethnicity, educational level, physical activity, smoke, HbA1c, failing kidneys, hypertension, marital status, PIR, drinking status, creatinine, total energy intake.

BMl, body mass index; HbA1c, glycated hemoglobin A1c; HDL-C, high density lipoprotein cholesterol; TC, total cholesterol; PIR, family poverty income ratio; 95% CI, 95% confidence interval.

**Table 5:** Association between log-transformed serum zinc level and triglyceride-glucose index using two-piece-wise regression models within the age <60 years group and the age ≥ 60 years group (All participants).

**Variable**	<**60 years (*****n*** = **1,065)**				≥**60 years (*****n*** = **545)**			
	**Crude model**		**Adjusted model**		**Crude model**		**Adjusted model**	
**Log-transformed serum zinc (**μ**g/dL)**	β **(95%CI)**	***P-*** **value**	β **(95%CI)**	***P-*** **value**	β **(95%CI)**	***P-*** **value**	β **(95%CI)**	***P-*** **value**
< 1.94	0.39 (−1.23~2.00)	0.63	−0.41 (−1.65~0.84)	0.50	0.20 (−1.71~2.10)	0.83	1.98 (0.62~3.35)	0.01
≥1.94	1.85 (0.54~3.16)	0.01	1.21 (0.49~1.94)	0.002	0.38 (−1.58~2.34)	0.70	−0.05 (−1.98~1.89)	0.95

Adjusted model: gender, BMI, HDL, TC, uric acid, diabetes, trouble sleeping, age, race and ethnicity, educational level, physical activity, smoke, HbA1c, failing kidneys, hypertension, marital status, PIR, drinking status, creatinine, total energy intake.

BMl, body mass index; HbA1c, glycated hemoglobin A1c; HDL-C, high density lipoprotein cholesterol; TC, total cholesterol; TyG, Triglyceride-glucose index; PIR, family poverty income ratio; 95% CI, 95% confidence interval.

And the RCS analysis was also applied to investigated the dose-response association between log-transformed serum zinc level and TyG with age ≥60 years and age <60 years. Figure 2 shows that in the fully adjusted analyses log-transformed serum zinc was related to level of TyG in a J shaped nonlinear manner (*P* for nonlinearity = 0.014) in age <60 years group (B), but in a inverted-L shaped nonlinear manner (*P* for nonlinearity < 0.001) in age ≥60 years group (C).

### 3.4 Stratified analyses

A stratified analysis was conducted according to age, gender, BMI and diabetes to determine whether there were differential effects in the association between log-transformed serum zinc and TyG in American adults (aged ≥20 years). The results demonstrated that no statistically significant interactions were identified in any of the subgroups after stratification by gender, BMI and diabetes in model 3 (Supplementary Table S2). However, when log-transformed serum zinc was transformed into a categorical variable, an interaction between log-transformed serum zinc and TyG was observed in individuals aged <60 years and those aged ≥60 years (*P* value for the likelihood ratio test for the interaction was *P* = 0.017; Table 6). The individuals with the log-transformed serum zinc quartile 3 (Q3) group (1.94–1.97 μg/dL) were used as the baseline reference. In the group of individuals aged <60 years, those with the Q4 group of log-transformed serum zinc (1.98–2.37 μg/dL) exhibited an adjusted β for TyG of 0.096 (95% CI 0.009–0.18; *P* < 0.05) in comparison to the Q3 group, after adjusting for the variables in Model 3. In the group of individuals aged ≥60 years, those with the Q1 group of log-transformed serum zinc levels (1.69–1.89 μg/dL) exhibited an adjusted β for TyG of −0.169 (95% CI −0.280 to −0.058; *P* < 0.05) in comparison to the Q3 group.

**Table 6:** Interactive effect of log-transformed serum zinc and triglyceride-glucose index in patients within the age <60 years group and the age ≥ 60 years group (All participants).

**Variable**	<**60 years (*****n*** = **1,065)**		≥**60 years (*****n*** = **545)**		***P* for interaction**
	β **(95%CI)**	***P*** **-value**	β **(95%CI)**	***P*** **-value**	
Log-transformed serum zinc (μg/dL)	0.301 (−0.190, 0.791)	0.215	0.968 (0.297, 1.639)	0.007	0.166
**Quartiles (log-transformed serum zinc (**μ**g/dL))**					
Q1(1.69-1.89)	0.070 (−0.028, 0.169)	0.151	−0.169 (−0.280,−0.058)	0.005	
Q2(1.90-1.93)	0.010 (−0.070, 0.089)	0.801	−0.001 (−0.111, 0.109)	0.985	
Q3(1.94-1.97)	0 (reference)		0 (reference)		0.017
Q4(1.98-2.37)	0.096 (0.009, 0.184)	0.033	0.049 (−0.069, 0.167)	0.39	

Adjusted model: gender, BMI, HDL, TC, uric acid, diabetes, trouble sleeping, age, race and ethnicity, educational level, physical activity, smoke, HbA1c, failing kidneys, hypertension, marital status, PIR, drinking status, creatinine, total energy intake.

BMl, body mass index; HbA1c, glycated hemoglobin A1c; HDL-C, high density lipoprotein cholesterol; TC, total cholesterol; TyG, Triglyceride-glucose index; PIR, family poverty income ratio; 95% CI, 95% confidence interval.

## 4 Discussion

In this cross-sectional analysis of US adults aged ≥ 20 years, using NHANES data from 2011 to 2016, we identified a positive association between log-transformed serum zinc levels and TyG. Across all models, the effect size for log-transformed serum zinc with TyG (β = 0.50) remains relatively consistent in Models 1, 2, and 3. Notably, we observed a J-shaped non-linear relationship between log-transformed serum zinc levels and TyG, with an inflection point at ~1.94 μg/dL. Furthermore, a statistically significant interaction was identified between log-transformed serum zinc levels and TyG in individuals aged ≥60 years and those <60 years (*P* < 0.05). These findings have significant clinical implications.

Insulin resistance has been suggested to play a noteworthy role in the pathogenesis of metabolic syndrome (30, 50, 51), and some evidences (51–54) suggests a direct association between serum zinc and insulin resistance which is consistent with our studies. For example, an 11-year prospective follow-up investigation was carried out among 683 male participants from the Kuopio Ischaemic Heart Disease Risk Factor Study (51) who were aged 42–60 years at baseline between 1984 and 1989. Teymoor Yary et al. (51) revealed that elevated serum zinc levels were linked to increased Homeostatic Model Assessment (HOMA) of insulin resistance and HOMA of beta cell. Additionally, a positive correlation was observed between higher serum zinc levels and the development of metabolic syndrome, as well as three of its constituent features, namely increased waist circumference, hypertension, and low serum HDL cholesterol (30). And a cross-sectional observational study (55) using NHANES data from 2011 to 2016 also revealed that serum zinc concentration was significantly higher in both abnormal glucose tolerance and diabetes mellitus groups when compared to the normal glucose tolerance group.

However, other studies have produced contrasting results. According to a cross-sectional study (56), it was found that the prevalence of insulin resistance (HOMA-IR; categorized according to the 75th percentile of the sample distribution) was elevated among Brazilian adolescents falling within the lower quartiles of zinc intake (< 7.5 mg), with a prevalence ratios (PR; 95% CI) of 1.23 (1.10–1.38) compared to those in the higher quartiles of zinc intake (>16.3 mg; *P* < 0.05).

One randomized, placebo-control study (57) found that zinc supplementation at 30 mg daily for 4 weeks significantly decreased fasting insulin and HOMA values in Brazilian obese women aged 25–45 years, but that plasma zinc, BMI, fasting glucose, and leptin levels were unaffected by zinc supplementation.

However, a study conducted in Korea (58) revealed that a daily zinc supplementation of 30 mg over an 8-week period enhanced serum zinc and urinary zinc concentrations in obese Korean women (BMI ≥25 kg/m^2^) aged 19–28 years. Nevertheless, the study found that zinc supplementation did not lead to improvements in insulin resistance (HOMA-IR) or in any other metabolic risk factors. Similarly, Beletate et al. (59) found that a 4-week zinc supplementation did not result in any significant improvements in insulin resistance, fasting glucose, or lipid levels in women who were obese and aged 25–45 years with normal glucose tolerance. Regina El Dib et al. (29) included three randomized controlled studies in their review. The duration of zinc supplementation ranged between four and 12 weeks. The trials' primary outcome measure was insulin resistance, which was assessed using the Homeostasis Model Assessment of Insulin Resistance (HOMA-IR). The comparative analysis of this parameter between the zinc supplemented cohort and the control group revealed no statistically significant disparities across two trials, which collectively enrolled 114 participants.

Table 3 indicates a significant association between the highest quartile of serum zinc and TyG. However, the underlying mechanisms remain unclear, with several potential mechanisms having been proposed. First, elevated zinc concentrations may influence hormonal homeostasis, including leptin. Such hormonal imbalances could potentially lead to an increase in BMI, which in turn may precipitate insulin resistance (53). Second, it has been demonstrated that zinc plays a significant role in the function of β-cells and the secretion of insulin (60). Consequently, it has been proposed that the activity of β-cells should be enhanced in order to facilitate the management of glucose levels among individuals with type 2 diabetes. However, excessive zinc intake may result in hyperactivity of β-cells and insulin production, potentially leading to insulin resistance through receptor exhaustion or prolonged zinc stimulation, which could have adverse effects on β-cells. Third, excessive zinc intake can also result in adverse effects, including altered copper and iron homeostasis, decreased concentrations of HDL cholesterol and serum lipoprotein, and impairment of liver function (61, 62). As previous studies have demonstrated that patients with type 2 diabetes have lower serum zinc concentrations and higher urinary zinc excretion compared to healthy controls (63, 64), this might be a protective mechanism aimed at eliminating surplus zinc to avert the onset of zinc-induced toxicity.

Very few studies have focused on relationship between serum zinc and TyG combined with age. In this study, the associations between serum zinc levels and TyG in participants aged ≥60 and <60 years were evaluated, adjusting for relevant variables. In Table 6, The results indicate a significant association between serum zinc and TyG in the ≥60 years group (β = 0.968, *P* = 0.007), but not in the <60 years group (β = 0.301, *P* = 0.215). However, the underlying mechanisms remain unclear. On the one hand, zinc plays a crucial role in immune function, and its deficiency is more prevalent in older adults, documented by a decline in serum or plasma zinc levels with age. Low zinc status is associated with a weakened immune system, but long-term and high-dose zinc supplementation may lead to some potential adverse effects, such as copper deficiency (65) and immunosuppressive, especially suppress T cell mediated events which will have a significant impact on the immunological outcome (66). This process may be associated with insulin resistance. On the other hand, in contrast to younger individuals, elderly patients experience a decline in physiological functions, making them more prone to various metabolic disorders (3). Abnormal bioelement levels can contribute to metabolic syndrome, especially in aging men. Zinc plays a crucial role in the function of beta cells within the islets of Langerhans. Animal studies indicate that low doses of zinc can protect against type 2 diabetes, whereas high concentrations can be toxic to these beta cells. This toxicity may lead to insulin resistance (34).

This study has several strengths. Firstly, the study encompasses a large, nationally representative sample of US adults. Secondly, the investigation modeled the associations between serum zinc and TyG while accounting for established and potential covariates. Thirdly, the study explored associations stratified by age groups of ≥60 and <60 years. Furthermore, a dose-response analysis was conducted to assess the relationship between serum zinc and TyG, as well as with age groups of ≥60 and <60 years.

Despite the strengths of the study, several limitations should be noted. First, this study was conducted with a US population, additional research is required to confirm whether our results can be generalized to other populations. Second, residual confounding effects could not be excluded. We constructed multivariable linear regression models and performed subgroup and sensitivity analyses to control for the effects of potential confounders on the relationship between serum zinc and TyG. Third, the study is in the lack of data on zinc intake in the population under investigation. High-quality, interventional and prospective studies are required to clarify the effects of zinc intake on TyG. Four, we recognize that nonrandom missing data could influence our findings due to baseline differences between included and excluded participants. To address this, we adopted a rigorous methodological approach. Following NHANES guidelines, we conducted a weighted analysis to account for survey design intricacies, including stratification and weighting, ensuring our results are representative of the broader U.S. population. Additionally, we performed model adjustments to enhance the reliability and robustness of our outcomes. Finally, because this was a cross-sectional observation study, the associations found in this study may not result in direct causality (67). Our study, a secondary analysis of publicly available data, explores the association between serum zinc and TyG index in adult Americans. While the evidence level from such secondary analyses is lower than that from primary studies, they effectively utilize existing data and can lay the groundwork for future research. Therefore, longitudinal studies are required to determine whether the observed relationship between the serum zinc and TyG is causal, as well as to explore the interactive effect of age on serum zinc and TyG. There may be a mechanistic association between age and TyG, which requires further investigation due to the biological distinctions it creates.

## 5 Conclusions

A J-shaped, non-linear positive correlation was observed between serum zinc levels and TyG, with an inflection point at ~1.94 μg/dL. Additionally, a statistically significant interaction was noted between serum zinc levels and TyG in individuals aged ≥60 years and those <60 years. In the age <60 years group, serum zinc exhibited a J-shaped non-linear association with TyG, while in the age ≥60 years group, the relationship followed an inverted-L shaped non-linear pattern. These outcomes suggest that there are potential adverse effects of high serum zinc levels on glucose metabolism by levels of TyG. Although this study offers valuable clinical insights, further prospective research is warranted to substantiate these findings, and to delve into the underlying mechanisms.

## Data Availability

NHANES data used in this work is publicly available. All raw data are available on the NHANES website (https://www.cdc.gov/nchs/nhanes/).

## References

[1] Seo JA Song SW Han K Lee KJ Kim HN. The associations between serum zinc levels and metabolic syndrome in the Korean population: findings from the 2010 Korean National Health and Nutrition Examination Survey. PLoS ONE. (2014) 9:e105990. 10.1371/journal.pone.0105990 25153887 PMC4143320

[2] Dubey P Thakur V Chattopadhyay M. Role of minerals and trace elements in diabetes and insulin resistance. Nutrients. (2020) 12:1864. 10.3390/nu12061864 32585827 PMC7353202

[3] Olechnowicz J Tinkov A Skalny A Suliburska J. Zinc status is associated with inflammation, oxidative stress, lipid, and glucose metabolism. J Physiol Sci. (2018) 68:19–31. 10.1007/s12576-017-0571-7 28965330 PMC5754376

[4] Maret W. Zinc in pancreatic islet biology, insulin sensitivity, and diabetes. Prev Nutr Food Sci. (2017) 22:1–8. 10.3746/pnf.2017.22.1.1 28401081 PMC5383135

[5] Lu CW Lee YC Kuo CS Chiang CH Chang HH Huang KC. Association of serum levels of zinc, copper, and iron with risk of metabolic syndrome. Nutrients. (2021) 13:548. 10.3390/nu13020548 33562398 PMC7914992

[6] Prasad AS. Clinical, endocrinological and biochemical effects of zinc deficiency. Clin Endocrinol Metab. (1985) 14:567–89. 10.1016/S0300-595X(85)80007-4 3905080

[7] Hong JS Park MK Kim MS Lim JH Park GJ Maeng EH. Effect of zinc oxide nanoparticles on dams and embryo-fetal development in rats. Int J Nanomed. (2014) 9:145–57. 10.2147/IJN.S57931 25565833 PMC4279755

[8] Wang B Feng W Wang M Wang T Gu Y Zhu M. Acute toxicological impact of nano- and submicro-scaled zinc oxide powder on healthy adult mice. J Nanoparticle Res. (2008) 10:263–76. 10.1007/s11051-007-9245-3

[9] Yan G Huang Y Bu Q Lv L Deng P Zhou J. Zinc oxide nanoparticles cause nephrotoxicity and kidney metabolism alterations in rats. J Environ Sci Health A Tox Hazard Subst Environ Eng. (2012) 47:577–88. 10.1080/10934529.2012.650576 22375541

[10] King JC Brown KH Gibson RS Krebs NF Lowe NM Siekmann JH. Biomarkers of Nutrition for Development (BOND)-zinc review. J Nutr. (2016) 146:858S–85S. 10.3945/jn.115.220079 26962190 PMC4807640

[11] Di Pino A DeFronzo RA. Insulin resistance and atherosclerosis: implications for insulin-sensitizing agents. Endocr Rev. (2019) 40:1447–67. 10.1210/er.2018-00141 31050706 PMC7445419

[12] Beverly JK Budoff MJ. Atherosclerosis: pathophysiology of insulin resistance, hyperglycemia, hyperlipidemia, and inflammation. J Diabetes. (2020) 12:102–4. 10.1111/1753-0407.12970 31411812

[13] Ding X Wang X Wu J Zhang M Cui M. Triglyceride-glucose index and the incidence of atherosclerotic cardiovascular diseases: a meta-analysis of cohort studies. Cardiovasc Diabetol. (2021) 20:76. 10.1186/s12933-021-01268-9 33812373 PMC8019501

[14] Zhang Q Xiao S Jiao X Shen Y. The triglyceride-glucose index is a predictor for cardiovascular and all-cause mortality in CVD patients with diabetes or pre-diabetes: evidence from NHANES 2001–2018. Cardiovasc Diabetol. (2023) 22:279. 10.1186/s12933-023-02030-z 37848879 PMC10583314

[15] Bello-Chavolla OY Almeda-Valdes P Gomez-Velasco D Viveros-Ruiz T Cruz-Bautista I Romo-Romo A. METS-IR, a novel score to evaluate insulin sensitivity, is predictive of visceral adiposity and incident type 2 diabetes. Eur J Endocrinol. (2018) 178:533–44. 10.1530/EJE-17-0883 29535168

[16] Borai A Livingstone C Ferns GA. The biochemical assessment of insulin resistance. Ann Clin Biochem. (2007) 44:324–42. 10.1258/000456307780945778 17594780

[17] Li S Ding J Sun X Feng L Zhou W Gui Z. Selenium concentration is positively associated with triglyceride-glucose index and triglyceride glucose-body mass index in adults: data from NHANES 2011–2018. Biol Trace Elem Res. (2024) 202:401–9. 10.1007/s12011-023-03684-2 37145256 PMC10764531

[18] Simental-Mendía LE Rodríguez-Morán M Guerrero-Romero F. The product of fasting glucose and triglycerides as surrogate for identifying insulin resistance in apparently healthy subjects. Metab Syndr Relat Disord. (2008) 6:299–304. 10.1089/met.2008.0034 19067533

[19] Tahapary DL Pratisthita LB Fitri NA Marcella C Wafa S Kurniawan F. Challenges in the diagnosis of insulin resistance: focusing on the role of HOMA-IR and tryglyceride/glucose index. Diabetes Metab Syndr. (2022) 16:102581. 10.1016/j.dsx.2022.102581 35939943

[20] Liu D Yang K Gu H Li Z Wang Y Wang Y. Predictive effect of triglyceride-glucose index on clinical events in patients with acute ischemic stroke and type 2 diabetes mellitus. Cardiovasc Diabetol. (2022) 21:280. 10.1186/s12933-022-01704-4 36510223 PMC9743618

[21] Minh HV Tien HA Sinh CT Thang DC Chen CH Tay JC. Assessment of preferred methods to measure insulin resistance in Asian patients with hypertension. J Clin Hypertens. (2021) 23:529–37. 10.1111/jch.14155 33415834 PMC8029536

[22] Park B Lee HS Lee YJ. Triglyceride glucose (TyG) index as a predictor of incident type 2 diabetes among nonobese adults: a 12-year longitudinal study of the Korean Genome and Epidemiology Study cohort. Transl Res. (2021) 228:42–51. 10.1016/j.trsl.2020.08.003 32827706

[23] Cui C Liu L Zhang T Fang L Mo Z Qi Y. Triglyceride-glucose index, renal function and cardiovascular disease: a national cohort study. Cardiovasc Diabetol. (2023) 22:325. 10.1186/s12933-023-02055-4 38017519 PMC10685637

[24] Gao S Ma W Huang S Lin X Yu M. Impact of triglyceride-glucose index on long-term cardiovascular outcomes in patients with Myocardial Infarction with nonobstructive coronary arteries. Nutr Metabolism Cardiovasc Dis. (2021) 31:3184–92. 10.1016/j.numecd.2021.07.027 34511291

[25] Tao L Xu J Wang T Hua F Li J. Triglyceride-glucose index as a marker in cardiovascular diseases: landscape and limitations. Cardiovasc Diabetol. (2022) 21:1–17. 10.1186/s12933-022-01511-x 35524263 PMC9078015

[26] Fernández-Cao JC Warthon-Medina M Hall Moran V Arija V Doepking C Lowe NM. Dietary zinc intake and whole blood zinc concentration in subjects with type 2 diabetes versus healthy subjects: a systematic review, meta-analysis and meta-regression. J Trace Elem Med Biol. (2018) 49:241–51. 10.1016/j.jtemb.2018.02.008 29452774

[27] Li YV. Zinc and insulin in pancreatic beta-cells. Endocrine. (2014) 45:178–89. 10.1007/s12020-013-0032-x 23979673

[28] Jansen J Karges W Rink L. Zinc and diabetes-clinical links and molecular mechanisms. J Nutr Biochem. (2009) 20:399–417. 10.1016/j.jnutbio.2009.01.009 19442898

[29] El Dib R Gameiro OL Ogata MS Módolo NS Braz LG Jorge EC. Zinc supplementation for the prevention of type 2 diabetes mellitus in adults with insulin resistance. Cochrane Database Syst Rev. (2015) 2015:CD005525. 10.1002/14651858.CD005525.pub3 26020622 PMC9939964

[30] Ghasemi A Zahediasl S Hosseini-Esfahani F Azizi F. Gender differences in the relationship between serum zinc concentration and metabolic syndrome. Ann Hum Biol. (2014) 41:436–42. 10.3109/03014460.2013.870228 24588511

[31] Yu Y Cai Z Zheng J Chen J Zhang X Huang X. Serum levels of polyunsaturated fatty acids are low in Chinese men with metabolic syndrome, whereas serum levels of saturated fatty acids, zinc, and magnesium are high. Nutr Res. (2012) 32:71–7. 10.1016/j.nutres.2011.12.004 22348454

[32] Pattan V Chang Villacreses MM Karnchanasorn R Chiu KC Samoa R. Daily intake and serum levels of copper, selenium and zinc according to glucose metabolism: cross-sectional and comparative study. Nutrients. (2021) 13:4044. 10.3390/nu13114044 34836302 PMC8622420

[33] Pushparani DS Anandan SN Theagarayan P. Serum zinc and magnesium concentrations in type 2 diabetes mellitus with periodontitis. J Indian Soc Periodontol. (2014) 18:187–93. 10.4103/0972-124X.131322 24872627 PMC4033885

[34] Rotter I Kosik-Bogacka D Dołegowska B Safranow K Lubkowska A Laszczyńska M. Relationship between the concentrations of heavy metals and bioelements in aging men with metabolic syndrome. Int J Environ Res Public Health. (2015) 12:3944–61. 10.3390/ijerph120403944 25867198 PMC4410226

[35] Obeid O Elfakhani M Hlais S Iskandar M Batal M Mouneimne Y. Plasma copper, zinc, and selenium levels and correlates with metabolic syndrome components of lebanese adults. Biol Trace Elem Res. (2008) 123:58–65. 10.1007/s12011-008-8112-0 18288450

[36] Mao Y Li X Zhu S Ma J Geng Y Zhao Y. Associations between urea nitrogen and risk of depression among subjects with and without type 2 diabetes: a nationwide population-based study. Front Endocrinol. (2022) 13:985167. 10.3389/fendo.2022.985167 36387890 PMC9646599

[37] Mao Y Li X Zhu S Geng Y. Association between dietary fiber intake and risk of depression in patients with or without type 2 diabetes. Front Neurosci. (2022) 16:920845. 10.3389/fnins.2022.920845 36389250 PMC9642095

[38] Yalcin G Ozsoy E Karabag T. The relationship of body composition indices with the significance, extension and severity of coronary artery disease. Nutr Metab Cardiovasc Dis. (2020) 30:2279–85. 10.1016/j.numecd.2020.07.014 32928627

[39] Matsunaga M Lim E Davis J Chen JJ. Dietary quality associated with self-reported diabetes, osteoarthritis, and rheumatoid arthritis among younger and older US adults: a cross-sectional study using NHANES 2011-2016. Nutrients. (2021) 13:545. 10.3390/nu13020545 33562353 PMC7915480

[40] Deng M Liu F Liang Y Chen Y Nie J Chai C. Associations of serum zinc, copper, and selenium with sleep disorders in the American adults: data from NHANES 2011-2016. J Affect Disord. (2023) 323:378–85. 10.1016/j.jad.2022.11.088 36464094

[41] Cheng Y Fang Z Zhang X Wen Y Lu J He S. Association between triglyceride glucose-body mass index and cardiovascular outcomes in patients undergoing percutaneous coronary intervention: a retrospective study. Cardiovasc Diabetol. (2023) 22:75. 10.1186/s12933-023-01794-8 36997935 PMC10064664

[42] Agricultural Research Service US US Department of Agriculture. What we eat in America: data tables. Available at: https://www.ars.usda.gov/northeast-area/beltsville-md-bhnrc/beltsville-human-nutrition-research-center/food-surveys-research-group/docs/wweia-data-tables/ (accessed August 3, 2022).

[43] Liu H Wang L Chen C Dong Z Yu S. Association between dietary niacin intake and migraine among american adults: national health and nutrition examination survey. Nutrients. (2022) 14:3052. 10.3390/nu14153052 35893904 PMC9330821

[44] Chobanian AV Bakris GL Black HR Cushman WC Green LA Izzo JL Jr. The seventh report of the joint national committee on prevention, detection, evaluation, and treatment of high blood pressure: the JNC 7 report. JAMA. (2003) 289:2560–72. 10.1001/jama.289.19.2560 12748199

[45] Huang W Ma X Liang H Li H Chen J Fang L. Dietary magnesium intake affects the association between serum vitamin D and type 2 diabetes: a cross-sectional study. Front Nutr. (2021) 8:763076. 10.3389/fnut.2021.763076 34901114 PMC8656460

[46] Ostojic SM. Dietary creatine and kidney function in adult population: NHANES 2017-2018. Food Sci Nutr. (2021) 9:2257–9. 10.1002/fsn3.2200 33841841 PMC8020933

[47] Zhu L Zhou B Zhu X Cheng F Pan Y Zhou Y. Association between body mass index and female infertility in the United States: data from National Health and Nutrition Examination Survey 2013-2018. Int J Gen Med. (2022) 15:1821–31. 10.2147/IJGM.S349874 35221716 PMC8865871

[48] Liu H Wang Q Dong Z Yu S. Dietary zinc intake and migraine in adults: a cross-sectional analysis of the National Health and Nutrition Examination Survey 1999-2004. Headache. (2023) 63:127–35. 10.1111/head.14431 36588459

[49] Kant R Verma V Patel S Chandra R Chaudhary R Shuldiner AR. Effect of serum zinc and copper levels on insulin secretion, insulin resistance and pancreatic β cell dysfunction in US adults: findings from the National Health and Nutrition Examination Survey (NHANES) 2011-2012. Diabetes Res Clin Pract. (2021) 172:108627. 10.1016/j.diabres.2020.108627 33333205

[50] Gill H Mugo M Whaley-Connell A Stump C Sowers J. The key role of insulin resistance in the cardiometabolic syndrome. Am J Med Sci. (2005) 330:290–4. 10.1097/00000441-200512000-00006 16355013

[51] Yary T Virtanen JK Ruusunen A Tuomainen TP Voutilainen S. Association between serum zinc and later development of metabolic syndrome in middle aged and older men: the Kuopio Ischaemic Heart Disease Risk Factor Study. Nutrition. (2017) 37:43–7. 10.1016/j.nut.2016.09.004 28359361

[52] Yary T Virtanen JK Ruusunen A Tuomainen TP Voutilainen S. Serum zinc and risk of type 2 diabetes incidence in men: the Kuopio ischaemic heart disease risk factor study. J Trace Elem Med Biol. (2016) 33:120–4. 10.1016/j.jtemb.2015.11.001 26653753

[53] Taneja SK Jain M Mandal R Megha K. Excessive zinc in diet induces leptin resistance in wistar rat through increased uptake of nutrients at intestinal level. J Trace Elem Med Biol. (2012) 26:267–72. 10.1016/j.jtemb.2012.03.002 22683053

[54] Qu X Yang H Yu Z Jia B Qiao H Zheng Y. Serum zinc levels and multiple health outcomes: implications for zinc-based biomaterials. Bioact Mater. (2020) 5:410–22. 10.1016/j.bioactmat.2020.03.006 32258830 PMC7114479

[55] Zhang J Hu J Zhao J Li J Cai X. Serum zinc concentrations and prediabetes and diabetes in the general population. Biol Trace Elem Res. (2022) 200:1071–7. 10.1007/s12011-021-02739-6 33931825

[56] Siqueira de Andrade MI Oliveira JS Leal VS Cabral PC Lira PIC. Independent predictors of insulin resistance in Brazilian adolescents: results of the study of cardiovascular risk in adolescents–Brazil. PLoS ONE. (2021) 16:e0246445. 10.1371/journal.pone.0246445 33561171 PMC7872259

[57] Marreiro DN Geloneze B Tambascia MA Lerário AC Halpern A Cozzolino SM. Effect of zinc supplementation on serum leptin levels and insulin resistance of obese women. Biol Trace Elem Res. (2006) 112:109–18. 10.1385/BTER:112:2:109 17028377

[58] Kim J Lee S. Effect of zinc supplementation on insulin resistance and metabolic risk factors in obese Korean women. Nutr Res Pract. (2012) 6:221–25. 10.4162/nrp.2012.6.3.221 22808346 PMC3395787

[59] Beletate V El Dib RP Atallah AN. Zinc supplementation for the prevention of type 2 diabetes mellitus. Cochrane Database Syst Rev. (2007) 24:CD005525. 10.1002/14651858.CD005525.pub2 17253560

[60] Rink L Gabriel P. Extracellular and immunological actions of zinc. Biometals. (2001) 14:367–83. 10.1023/A:1012986225203 11831466

[61] Trumbo P Yates AA Schlicker S Poos M. Dietary reference intakes: vitamin A, vitamin K, arsenic, boron, chromium, copper, iodine, iron, manganese, molybdenum, nickel, silicon, vanadium, and zinc. J Am Diet Assoc. (2001) 101:294–301. 10.1016/S0002-8223(01)00078-5 11269606

[62] Hu J Cai X Li J Zheng N Zhang J. Associations between serum zinc levels and alanine aminotransferase elevation in adults. Biol Trace Elem Res. (2021) 199:2077–84. 10.1007/s12011-020-02318-1 32737810

[63] Al-Maroof RA Al-Sharbatti SS. Serum zinc levels in diabetic patients and effect of zinc supplementation on glycemic control of type 2 diabetics. Saudi Med J. (2006) 27:344–50. 16532095

[64] Carneiro G Laferrère B Zanella MT. Vitamin and mineral deficiency and glucose metabolism-a review. E-SPEN J. (2013) 8:e73–9. 10.1016/j.clnme.2013.03.003

[65] Calder PC Ortega EF Meydani SN Adkins Y Stephensen CB Thompson B. Nutrition, immunosenescence, and infectious disease: an overview of the scientific evidence on micronutrients and on modulation of the gut microbiota. Adv Nutr. (2022) 13:S1–26. 10.1093/advances/nmac052 36183242 PMC9526826

[66] Haase H Mocchegiani E Rink L. Correlation between zinc status and immune function in the elderly. Biogerontology. (2006) 7:421–8. 10.1007/s10522-006-9057-3 16953331

[67] Xiao Y Xiao Z. Association between serum Klotho and kidney stones in US middle-aged and older individuals with diabetes mellitus: results from 2007-2016 National Health and Nutrition Survey. Am J Nephrol. (2023) 54:224–33. 10.1159/000531045 37231844 PMC10614277

