# Supplementary material for: Age-dependent interaction between serum zinc and triglyceride-glucose index among American adults: National Health and Nutrition Examination Survey
Source: Front Nutr. 2025 Jan 13;11:1475204. doi: 10.3389/fnut.2024.1475204 (PMC11769825; doi:10.3389/fnut.2024.1475204)
Supplement: Supplementary file 1 [file Table_1.doc]

**Supplementary material**

**Table S1. Basic characteristics of excluded and included participants.**

| **Characteristic** | Total  (n = 31126) | Excluded population  (n = 20038) | Included population  (n = 11088) |
| --- | --- | --- | --- |
| Age ( Mean ± SD, years) | 31.6 ± 24.6 | 30.6 ± 24.5 | 49.6 ± 17.6 |
| Gender, n(%) |  |  |  |
| Male | 14751 (49.3) | 13912 (49.2) | 839 (52.1) |
| Female | 15151 (50.7) | 14380 (50.8) | 771 (47.9) |
| Race/Ethnicity, n(%) |  |  |  |
| Non-Hispanic White | 9713 (32.5) | 9017 (31.9) | 696 (43.2) |
| Non-Hispanic Black | 7079 (23.7) | 6776 (24) | 303 (18.8) |
| Mexican American | 5006 (16.7) | 4787 (16.9) | 219 (13.6) |
| Others | 8104 (27.1) | 7712 (27.3) | 392 (24.4) |
| Education level, n(%) |  |  |  |
| <9 | 1693 ( 9.9) | 1556 (10.1) | 137 (8.5) |
| 9-12 | 5957 (35.0) | 5401 (35) | 556 (34.5) |
| >12 | 9381 (55.1) | 8464 (54.9) | 917 (57.0) |
| Marital status, n(%) |  |  |  |
| Living alone | 9946 (58.4) | 8977 (58.2) | 969 (60.2) |
| Married or living with a partner | 7089 (41.6) | 6448 (41.8) | 641 (39.8) |
| PIR, n(%) |  |  |  |
| low (PIR ≤ 1.3) | 10844 (39.8) | 10318 (40.3) | 526 (32.7) |
| medium (PIR > 1.3 to 3.5) | 9449 (34.7) | 8845 (34.5) | 604 (37.5) |
| high (PIR > 3.5) | 6932 (25.5) | 6452 (25.2) | 480 (29.8) |
| BMI (kg/m^2^), Mean ± SD | 25.7 ± 7.9 | 25.4 ± 7.9 | 29.3 ±7.1 |
| BMI, n(%) |  |  |  |
| <18.5 kg/m^2^ | 5356 (20.3) | 5326 (21.5) | 30 (1.9) |
| 18.5-24.9 kg/m^2^ | 8001 (30.3) | 7559 (30.5) | 442 (27.4) |
| 25-29.9 kg/m^2^ | 6218 (23.5) | 5692 (22.9) | 526 (32.7) |
| ≥30 kg/m^2^ | 6838 (25.9) | 6226 (25.1) | 612 (38.0) |
| Physical activity, n(%) |  |  |  |
| Sedentary | 10063 (48.2) | 9320 (48.4) | 743 (46.2) |
| Moderate | 7331 (35.1) | 6794 (35.3) | 537 (33.4) |
| Vigorous | 3485 (16.7) | 3155 (16.4) | 330 (20.5) |
| Smoking status, n (%) |  |  |  |
| Never | 10275 (58.2) | 9385 (58.5) | 890 (55.3) |
| Former | 3928 (22.3) | 3520 (22) | 408 (25.3) |
| Current | 3440 (19.5) | 3128 (19.5) | 312 (19.4) |
| Drinking status, n (%) |  |  |  |
| ≥ 12 alcohol drinks a year | 10844 (69.5) | 9655 (69.1) | 1189 (73.8) |
| Trouble sleeping, n (%) | 4591 (24.2) | 4160 (24) | 431 (26.8) |
| HbA1c (%), Mean ± SD | 5.7 ± 1.1 | 5.7 ± 1.1 | 5.8 ± 1.1 |
| HDL-C (mg/dL), Mean ± SD | 53.4 ± 15.3 | 53.3 ± 15.3 | 54.0 ± 15.8 |
| TG (mg/dL), Mean ± SD | 113.0 ± 100.6 | 112.7 ± 106.8 | 114.1 ± 65.4 |
| Creatinine (mg/dL), Mean ± SD | 0.89 ± 0.45 | 0.84 ± 0.36 | 0.89 ± 0.45 |
| TC (mg/dL), Mean ± SD | 180.9 ± 41.3 | 180.2 ± 41.2 | 190.9 ± 40.7 |
| Energy (kcal), Mean ± SD | 1968.7 ± 955.5 | 1958.5 ± 960.1 | 2119.6 ± 873.2 |
| FPG (mg/dL), Mean ± SD | 107.1 ± 34.7 | 106.8 ± 34.8 | 108.7 ± 34.0 |
| Uric acid (mg/dL), Mean ± SD | 5.3 ± 1.4 | 5.3 ± 1.4 | 5.5 ± 1.4 |
| TyG, Mean ± SD | 8.5 ± 0.7 | 8.5 ± 0.7 | 8.6 ± 0.6 |
| log-transformed serum zinc (μg/dL), Mean ± SD | 1.9 ± 0.1 | 1.9 ± 0.1 | 1.9 ± 0.1 |
| Hypertension, n (%) | 8099 (27.1) | 7351 (26) | 748 (46.5) |
| Diabetes, n (%) | 3255 (34.6) | 2890 (37.1) | 365 (22.7) |
| Failing kidneys, n (%) | 626 ( 3.7) | 566 (3.7) | 60 (3.7) |

Abbreviations: SD, standard deviation; PIR, family poverty income ratio; BMl, body mass index; HbA1c, glycated hemoglobin A1c; HDL-C, high density lipoprotein cholesterol; TG, triglyceride; TC, total cholesterol; FPG, fasting blood glucose; TyG, Triglyceride-glucose index.

**Table S2 Stratified multivariable analysis of the association between log-transformed serum zinc levels and TyG (all participants).**

| **Variable** | **β (95%CI)** | **P value** | **P for interaction** |
| --- | --- | --- | --- |
| **Gender** |  |  | 0.599 |
| **Male** |  |  |  |
| Quartiles (log-transformed serum zinc (μg/dL)) |  |  |  |
| Q1(1.69-1.89) | -0.018 (-0.127, 0.090) | 0.73 |  |
| Q2(1.90-1.93) | 0.030 (-0.051, 0.111) | 0.45 |  |
| Q3(1.94-1.97) | Reference |  |  |
| Q4(1.98-2.37) | 0.060 (-0.026, 0.146) | 0.16 |  |
| P for Trend |  | 0.09 |  |
| **Female** |  |  |  |
| Quartiles (log-transformed serum zinc (μg/dL)) |  |  |  |
| Q1(1.69-1.89) | 0.024 (-0.074, 0.122) | 0.62 |  |
| Q2(1.90-1.93) | 0.002 (-0.100, 0.105) | 0.96 |  |
| Q3(1.94-1.97) | Reference |  |  |
| Q4(1.98-2.37) | 0.116 (0.014, 0.218) | 0.03 |  |
| P for Trend |  | 0.07 |  |
| **Age (years)** |  |  | 0.017 |
| **<60** |  |  |  |
| Quartiles (log-transformed serum zinc (μg/dL)) |  |  |  |
| Q1(1.69-1.89) | 0.070 (-0.028, 0.169) | 0.15 |  |
| Q2(1.90-1.93) | 0.010 (-0.070, 0.089) | 0.8 |  |
| Q3(1.94-1.97) | Reference |  |  |
| Q4(1.98-2.37) | 0.096 (0.009, 0.184) | 0.03 |  |
| P for Trend |  | 0.08 |  |
| **≥60** |  |  |  |
| Quartiles (log-transformed serum zinc (μg/dL)) |  |  |  |
| Q1(1.69-1.89) | -0.169 (-0.280, -0.058) | 0.01 |  |
| Q2(1.90-1.93) | -0.001 (-0.111, 0.109) | 0.99 |  |
| Q3(1.94-1.97) | Reference |  |  |
| Q4(1.98-2.37) | 0.049 (-0.069, 0.167) | 0.39 |  |
| P for Trend |  | 0.11 |  |
| **BMI ( kg/m^2^)** |  |  | 0.621 |
| **<18.5 kg/m^2^** |  |  |  |
| Quartiles (log-transformed serum zinc (μg/dL)) |  |  |  |
| Q1(1.69-1.89) | 0.233 (NaN, NaN) |  |  |
| Q2(1.90-1.93) | 0.350 (NaN, NaN) |  |  |
| Q3(1.94-1.97) | Reference |  |  |
| Q4(1.98-2.37) | 0.452 (NaN, NaN) |  |  |
| P for Trend |  |  |  |
| **18.5-24.9 kg/m^2^** |  |  |  |
| Quartiles (log-transformed serum zinc (μg/dL)) |  |  |  |
| Q1(1.69-1.89) | 0.041 (-0.132, 0.214) | 0.62 |  |
| Q2(1.90-1.93) | 0.071 (-0.047, 0.188) | 0.22 |  |
| Q3(1.94-1.97) | Reference |  |  |
| Q4(1.98-2.37) | 0.091 (-0.048, 0.230) | 0.18 |  |
| P for Trend |  | 0.13 |  |
| **25-29.9 kg/m^2^** |  |  |  |
| Quartiles (log-transformed serum zinc (μg/dL)) |  |  |  |
| Q1(1.69-1.89) | 0.038 (-0.107, 0.183) | 0.58 |  |
| Q2(1.90-1.93) | -0.018 (-0.116, 0.080) | 0.7 |  |
| Q3(1.94-1.97) | Reference |  |  |
| Q4(1.98-2.37) | 0.097 (-0.025, 0.219) | 0.11 |  |
| P for Trend |  | 0.19 |  |
| **≥30 kg/m^2^** |  |  |  |
| Quartiles (log-transformed serum zinc (μg/dL)) |  |  |  |
| Q1(1.69-1.89) | -0.035 (-0.141, 0.072) | 0.5 |  |
| Q2(1.90-1.93) | -0.004 (-0.122, 0.114) | 0.94 |  |
| Q3(1.94-1.97) | Reference |  |  |
| Q4(1.98-2.37) | 0.109 (-0.003, 0.221) | 0.06 |  |
| P for Trend |  | 0.07 |  |
| **Diabetes** |  |  | 0.755 |
| **No** |  |  |  |
| Quartiles (log-transformed serum zinc (μg/dL)) |  |  |  |
| Q1(1.69-1.89) | 0.034 (-0.058, 0.126) | 0.44 |  |
| Q2(1.90-1.93) | 0.027 (-0.042, 0.095) | 0.42 |  |
| Q3(1.94-1.97) | Reference |  |  |
| Q4(1.98-2.37) | 0.096 (0.002, 0.191) | 0.05 |  |
| P for Trend |  | 0.04 |  |
| **Yes** |  |  |  |
| Quartiles (log-transformed serum zinc (μg/dL)) |  |  |  |
| Q1(1.69-1.89) | -0.095 (-0.299, 0.109) | 0.32 |  |
| Q2(1.90-1.93) | -0.047 (-0.265, 0.171) | 0.64 |  |
| Q3(1.94-1.97) | Reference |  |  |
| Q4(1.98-2.37) | 0.088 (-0.142, 0.318) | 0.41 |  |
| P for Trend |  | 0.36 |  |

Adjusted for gender, BMI, HDL, TC, uric acid, diabetes, trouble sleeping, age, race and ethnicity, educational level, physical activity, smoke, HbA1c, failing kidneys, hypertension, marital status, PIR, drinking status, creatinine, total energy intake.

Abbreviations: BMl, body mass index; 95% CI, 95% confidence interval.
